# Schiller as a Physician

**Published:** 1860-05

**Authors:** 


### Editors' Table.

Schiller as a Physician.—We are indebted to Dr. Winternitz, of Vienna, one of the contributors to our esteemed contemporary the "*Oesterreichische Zeitschrift für Praktische Heilkunde*, for a very interesting and novel account of "Schiller Considered as a Physician," written on the occasion of the anniversary of his one hundredth birth-day. It is so rarely that this great author is spoken of in a medical capacity that those who have regarded him merely as one of the greatest of tragic and dramatic writers will be glad to have an outline of the article before us.

Schiller commenced the study of medicine in 1775, at Stuttgard, in the academy erected by the Duke of Wurtemberg for the benefit of his officers' sons. For two years he pursued his medical researches with great zest, and wrote a Latin treatise on the "Philosophy of Physiology," which was never printed; in 1780 he composed his essay on the "Connection of the Animal and Spiritual Natures of Man," which was published in 1821 in the *Monatschrift*, of Berlin. In the same year he received the appointment of surgeon to the regiment Augé, in the Wurtemberg army, and performed his duties most satisfactorily and with great fidelity.

Schiller's intimate friend, Scharffenstein, has transmitted to us a ludicrous description of the young surgeon as he appeared on parade in the full glory of his regimentals. His body was squeezed into a uniform cut in the old Prussian style; on each side of his lace were three stiff rolls intended to represent curls; a heavy cue was surmounted by a little military hat, which scarcely covered the crown of his head, and his long neck protruded from a tight horse-hair stock. But the most wonderful part of this costume was the foot and leg attire; the feet were forced into gaiters covered with felt, and two cylinders of great size were compressed into the narrow stockings which were scarcely able to incase his legs, and certainly no additional encumbrances.

Schiller studied medicine rather from compulsion than choice, and finding that it seriously interfered with his literary labors, he determined to abandon it altogether. This course seems to have been forced upon him by the Duke, who, after the publication of the "Robbers," sent for Schiller, and ordered him to confine his writings to medical subjects. Feeling this to be an impossibility, Schiller l[illegible]t his regiment, in consequence of which he was banished from Stuttgard, and compelled to live under an assumed name in the adjacent towns.

Considering his reputation as a poet, dramatist, historian, and philosopher, we can scarcely regret Schiller's withdrawal from our ranks; for, though it may have been our loss in a medical point of view, we have certainly been more than compensated by the varied emanations of his wonderful genius.

Carlyle tells us that during the last fifteen years of his life, Schiller's sufferings were intense, and that on a surgical inspection of his body after death, the most vital organs were found deranged. "The structure of the lungs was in great part destroyed; the cavities of the heart were nearly grown up; the liver had become hard, and the gall-bladder was extended to an extraordinary size."

